# Evaluation of canine adipose-derived mesenchymal stem cells for neurological functional recovery in a rat model of traumatic brain injury

**DOI:** 10.1186/s12917-024-03912-4

**Published:** 2024-03-18

**Authors:** Wenkang Jiang, Huina Luo, Mingming Zhao, Quanbao Fan, Cailing Ye, Xingying Li, Jing He, Jianyi Lai, Shi He, Wojun Chen, Weihang Xian, Shengfeng Chen, Zhisheng Chen, Dongsheng Li, Ruiai Chen, Bingyun Wang

**Affiliations:** 1 Zhaoqing Branch Center of Guangdong Laboratory for Lingnan Modern Agricultural Science and Technology, Zhaoqing, 526000 China; 2 School of Life Science and Engineering, Foshan University, Foshan, 528225 China; 3 Deja Lab, VetCell Biotechnology Company Limited, Foshan, 528225 China

**Keywords:** Traumatic brain injury, Adipose-derived mesenchymal stem cells, Therapy, Microglia, Attenuating inflammation

## Abstract

**Background:**

Traumatic brain injury (TBI) is a common condition in veterinary medicine that is difficult to manage.Veterinary regenerative therapy based on adipose mesenchymal stem cells seem to be an effective strategy for the treatment of traumatic brain injury. In this study, we evaluated therapeutic efficacy of canine Adipose-derived mesenchymal stem cells (AD-MSCs)in a rat TBI model, in terms of improved nerve function and anti-neuroinflammation.

**Results:**

Canine AD-MSCs promoted neural functional recovery, reduced neuronal apoptosis, and inhibited the activation of microglia and astrocytes in TBI rats. According to the results in vivo, we further investigated the regulatory mechanism of AD-MSCs on activated microglia by co-culture in vitro. Finally, we found that canine AD-MSCs promoted their polarization to the M2 phenotype, and inhibited their polarization to the M1 phenotype. What’s more, AD-MSCs could reduce the migration, proliferation and Inflammatory cytokines of activated microglia, which is able to inhibit inflammation in the central system.

**Conclusions:**

Collectively, the present study demonstrates that transplantation of canine AD-MSCs can promote functional recovery in TBI rats via inhibition of neuronal apoptosis, glial cell activation and central system inflammation, thus providing a theoretical basis for canine AD-MSCs therapy for TBI in veterinary clinic.

## Background

Traumatic brain injury (TBI), which is a common disease in dogs with a mortality range from 18–34% [1], is induced by external forces [2], resulting in structural damage and physiological disturbance of the brain. TBI has a wide variety of external symptoms, such as ataxia or dystaxia, ocular motor dysfunction, and disequilibrium and pathological changes in the central nervous system including neuronal and glial cell damage, inflammation, brain edema, and increased intracranial pressure [3]. Current therapies that mainly included surgery, hyperbaric oxygen, mild hypothermia, and hypertonic saline can only slightly relieve secondary reactions, but cannot effectively reduce neuronal apoptosis [4]. Therefore, it is highly desirable to develop an effective therapeutic strategy against TBI in dogs.

Mesenchymal stem cells (MSCs) are self-renewing precursor cells that possess multipotent ability to differentiate into various type of tissue cells on providing appropriate niche [5]. AD-MSCs have become an attractive approach for the treatment of traumatic brain injury due to their easy access, low immunogenicity, and high self-repair ability [6–8]. Recently, studies have shown that AD-MSCs have many positive effects on the treatment of TBI, including their ability to differentiate into various types of nerve cells [9], reduce neuronal death [10], inhibit inflammation in the brain [11], inhibit astroglial proliferation [11], and repair the blood-brain barrier (BBB) [12]. However, a few studies have simultaneously focused on the effects of canine AD-MSCs on neurons and glial cells in experimental TBI models. Herein, we therefore investigated the potential therapeutic effects and mechanisms of canine AD-MSCs in a model of TBI disease in rats.

## Results

### AD-MSCs promoted functional recovery in TBI-modeled rats

**Fig. 1:**
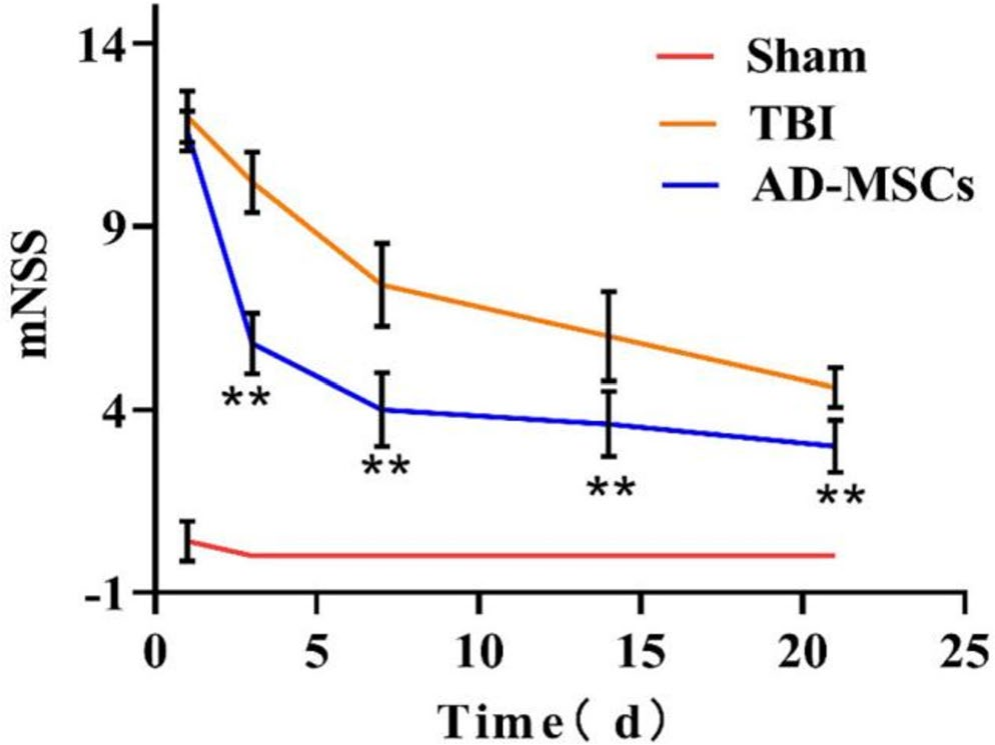
Modified neurological severity score. **indicates *p* < 0.01; *indicates *p* < 0.05

As shown in Fig. 1, the modified neurological severity score (mNSS) of the rats in the AD-MSCs group were remarkably lower than those in the TBI group at 3, 7, 14, and 21 d after modeling (*P* < 0.01), and were significantly higher than those in the Sham group (*P* < 0.01), indicating that AD-MSCs have the ability to improve the sensory and motor function of the TBI-modeled rats.

### AD-MSCs reduced inflammation levels in the blood of TBI rats

**Fig. 2:**
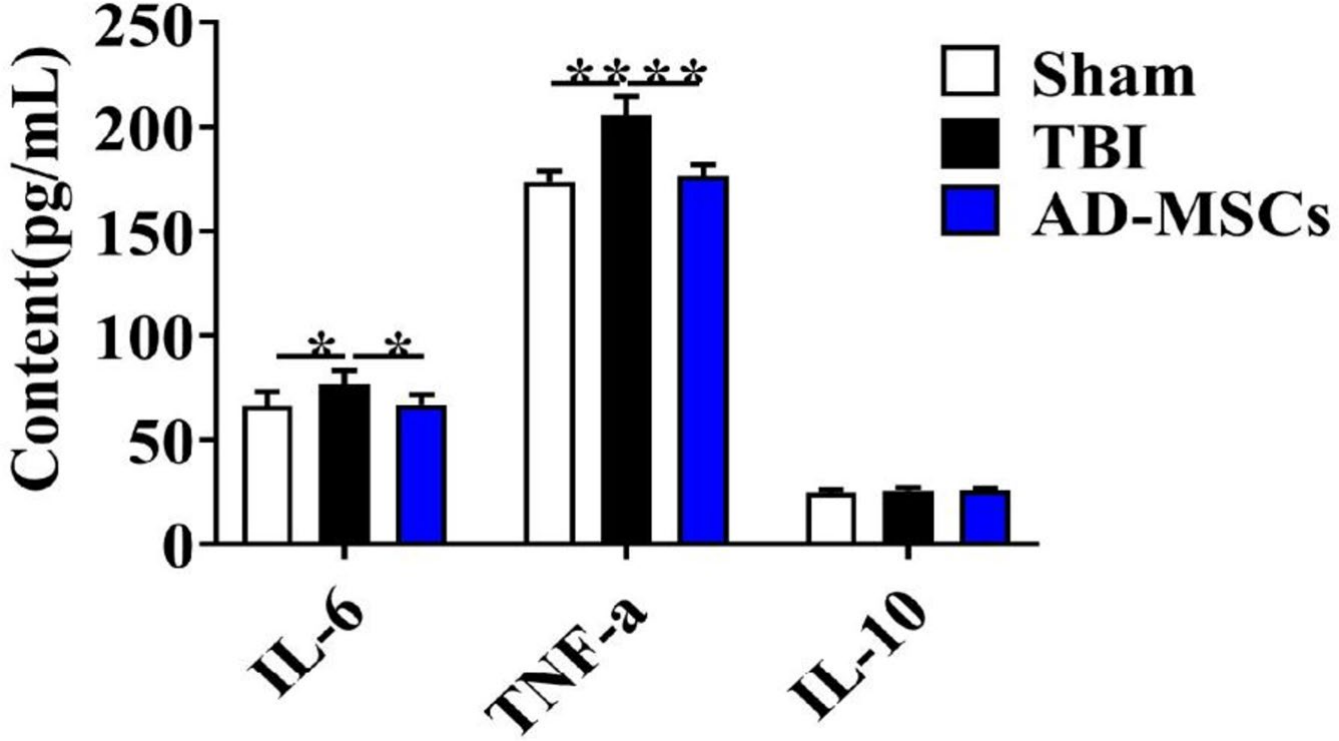
Serum inflammatory factors of rats on the 3rd day were detected by ELISA. **indicates *p* < 0.01; *indicates *p* < 0.05

As shown in Fig. 2, markedly decreased IL-6 release was observed at 3 d in the AD-MSCs group (*P* < 0.05) compared with TBI group. For the release of TNF-α, AD-MSCs significantly down-regulated the serum TNF-α level on the 3rd day of treatment (*P* < 0.01), as compared with TBI group. However, there was no significant difference in levels of the cytokines IL-10 in the serum of the 3 groups of rats (*P* > 0.05).

### AD-MSCs suppressed the activation of astrocytes and microglia in the injured brain

**Fig. 3:**
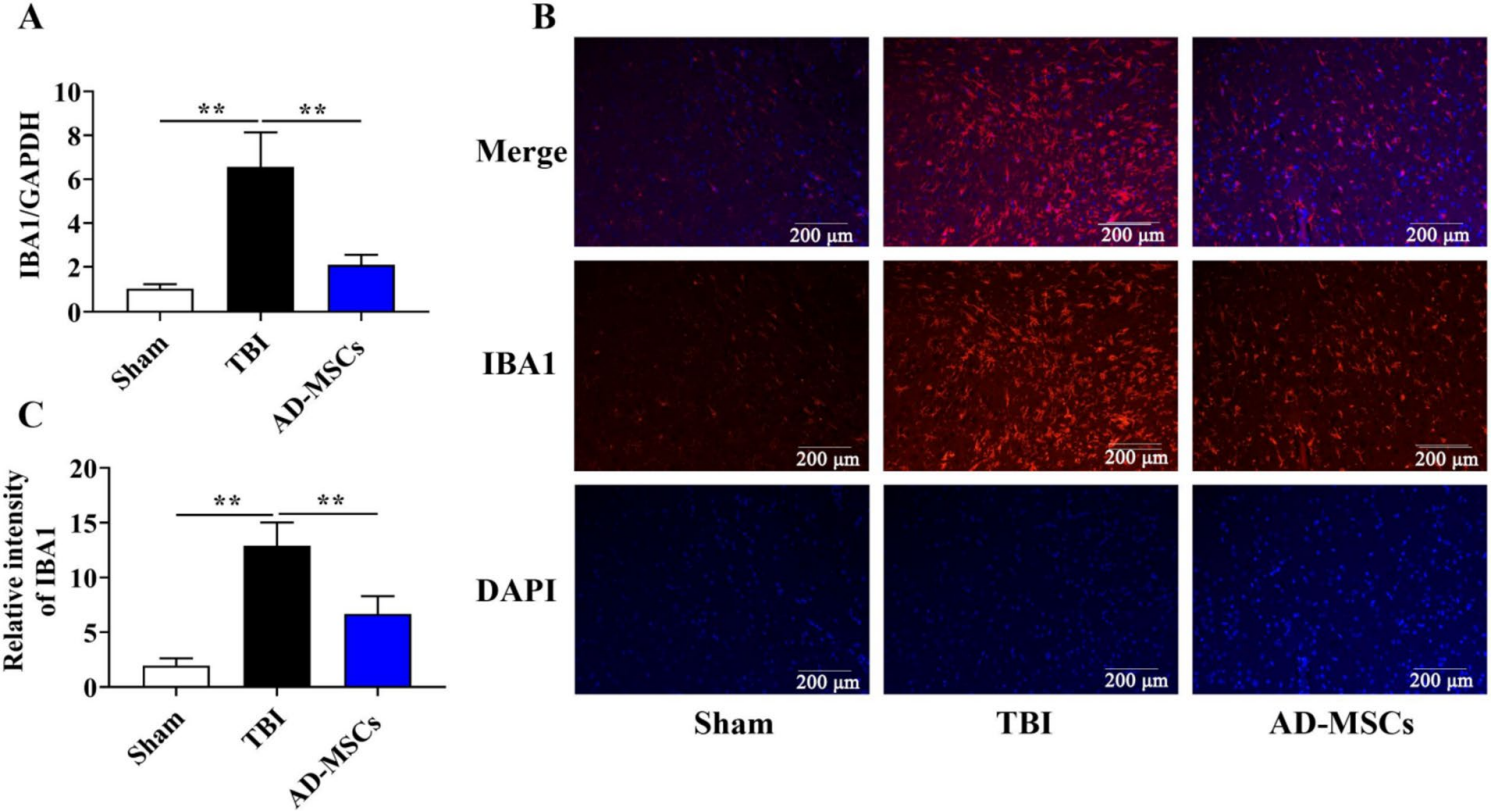
AD-MSCs inhibits the expression of IBA1. **A** The mRNA expression of microglia marker IBA1 in brain tissue was detected by qPCR; **B** The localized expression of microglia marker IBA1 protein was detected by immunofluorescence; **C** Relative intensity of IBA1. **indicates *p* < 0.01; *indicates *p* < 0.05. Scale bar is 200 μm

**Fig. 4:**
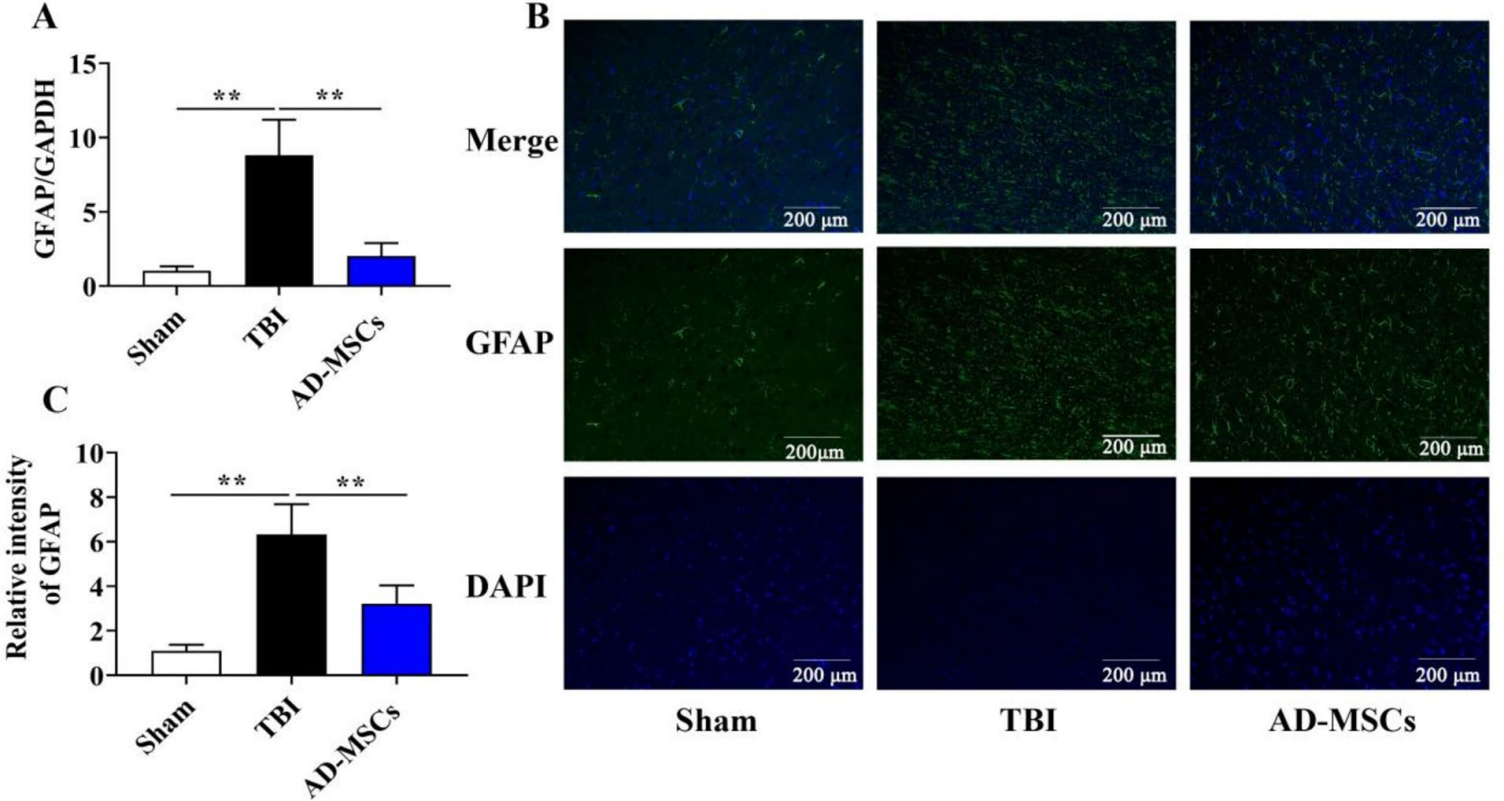
AD-MSCs inhibits the expression of GFAP. **A** The mRNA expression of astrocytemarker GFAP in brain tissue was detected by qPCR; **B** The localized expression ofastrocyte marker GFAP protein was detected by immunofluorescence; **C** Relative intensity of GFAP. **indicates *p* < 0.01; *indicates *p* < 0.05. Scale bar is 200 μm

Because the AD-MSCs exhibited modulation of immune responses in the TBI-modeled animals, the status of astrocytes and microglial cells, at 21 d post-injury was probed by staining with antibodies against glial fibrillary acidic portein (GFAP) for astrocytes and ionic calcium binds adapter molecule 1 (IBA1) for activated microglia. Our results from RT-qPCR showed that mRNA expressions of the IBA1 (Fig. 3A, *P* < 0.01) and GFAP (Fig. 4A, *P* < 0.01) in the AD-MSCs group were significantly lower than those in the TBI group. Furthermore, the fluorescence expression intensity of IBA1 protein (Fig. 3B and C, *P* < 0.01) and GFAP protein (Fig. 4B and C, *P* < 0.01) was also remarkably lower than those in the TBI group after 21 d of modeling. All together, these results highlight that the activation of glial cells in the animal brain was remarkably decreased after the treatment of AD-MSCs, indicating its beneficial role in neural repair.

### AD-MSCs limited the apoptosis of neural cells

**Fig. 5:**
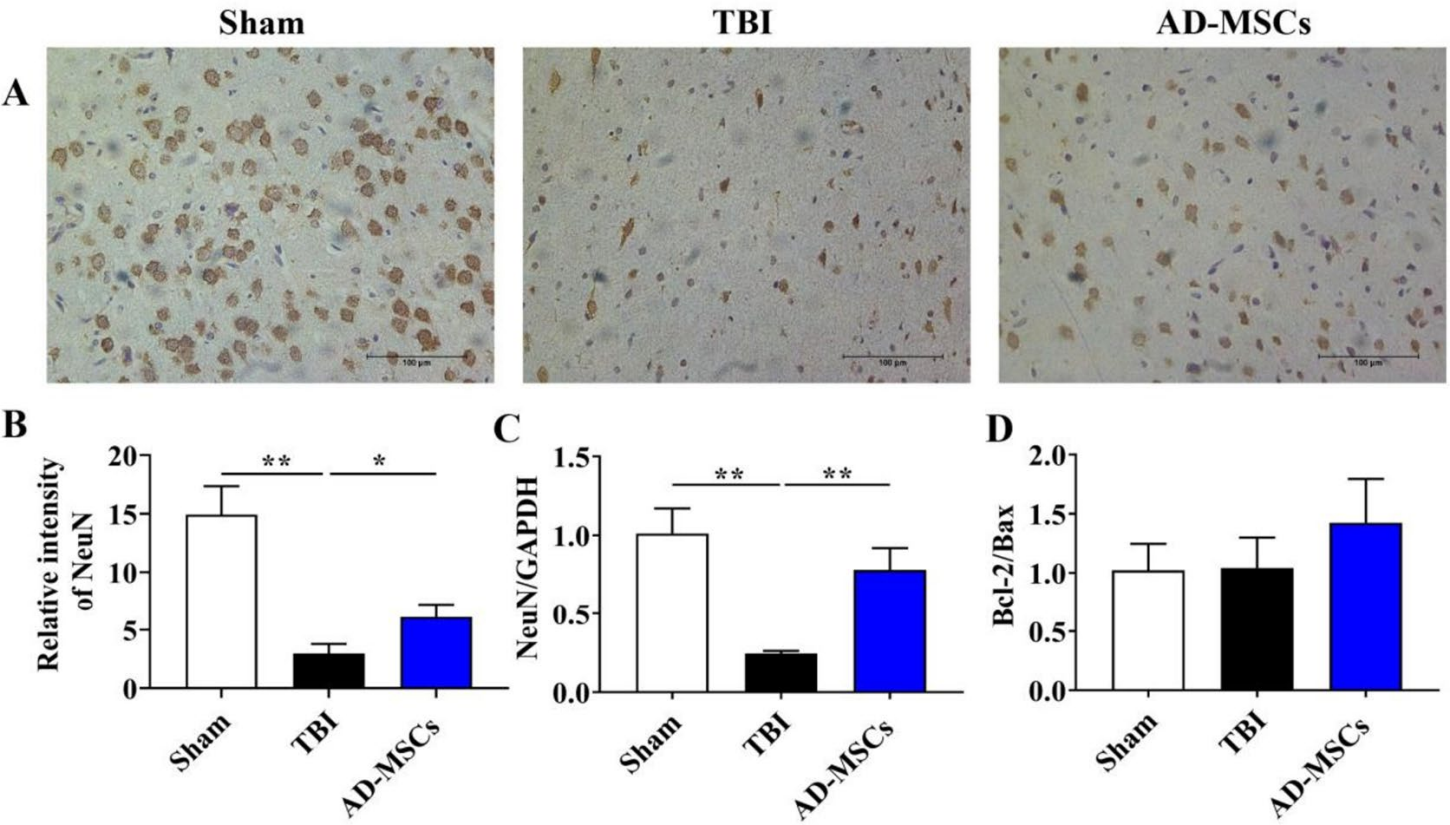
AD-MSCs limited the apoptosis of neural cells. **A** The localized expression of neural cells marker NeuN protein was detected by immunohistochemistry; **B** Relative intensity of NeuN. **C** The mRNA expression of neural cells marker NeuN in brain tissue was detected by qPCR. **D** The expression of Bcl2/Bax apoptosis-related genes was detected by qPCR. **indicates *p* < 0.01; *indicates *p* < 0.05.Scale bar is 100 μm

Levels of neuronal nuclei (NeuN), Bax/Bcl-2 reflect the apoptosis of neural cells. To test the effects of AD-MSCs treatment on the apoptosis of neural cells in the injured brain, we investigated the protein level of NeuN by immunohistochemistry and the mRNA level of NeuN and Bax/Bcl-2 by qPCR. Compared to rats of the TBI group, AD-MSCs treated group showed higher levels of NeuN protein on the 21st day of treatment (Fig. 5A and B, *P* < 0.05). RT-qPCR results showed that the expression of NeuN in AD-MSCs group was significantly higher than that in the TBI group at 21 d of modeling (Fig. 5C, *P* < 0.01). Furthermore as shown in Fig. 5D, the level of Bcl-2/Bax was increased after AD-MSCs intervention, reflecting that AD-MSCs reduced neuronal apoptosis. Altogether these results indicate that AD-MSCs decreased the apoptosis of neural cells in the injured brain.

### AD-MSCs attenuated TBI-induced brain tissue damage

**Fig. 6:**
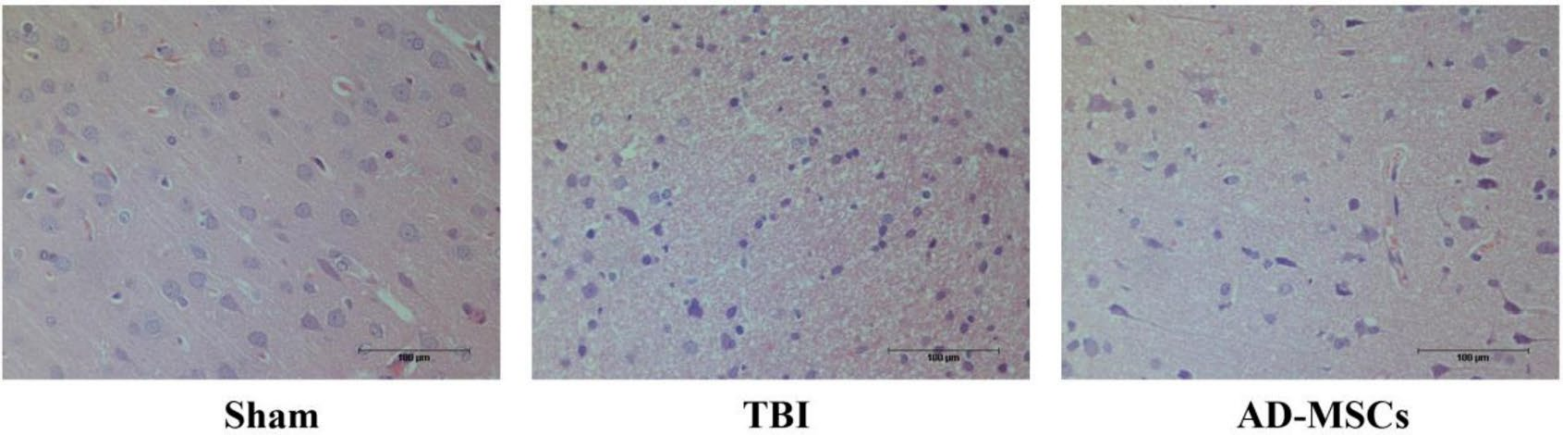
HE staining of rat brain tissue. Scale bar is 100 μm

As shown in Fig. 6, the brain tissue structure of the rats in the Sham group was normal, and that of the TBI-modeled rats showed typical pathological changes. Compared with the TBI group, the HE slices of the AD-MSCs treatment group showed that AD-MSCs could significantly reduce the area of brain tissue damage, edema, neuronal apoptosis, and the number of activated glial cells was also reduced (Fig. 6).

### AD-MSCs reduced the proliferation of lipopolysaccharide(LPS)-activated BV2 cells

**Fig. 7:**
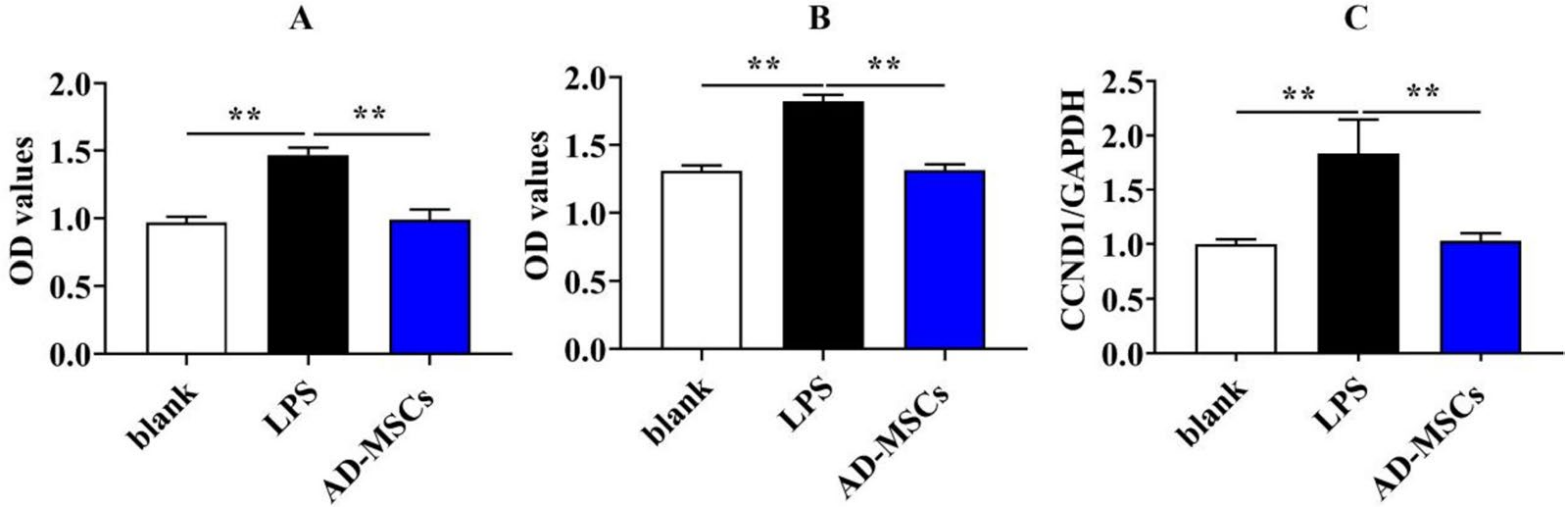
AD-MSCs reduced the proliferation of LPS-activated BV2 cells. **A**-**B** The effects of canine AD-MSCs on the proliferation of activated BV2 cells after 24 and 48 h were detected by microplate reader. **C** The mRNA expression of CCND1 genes of BV2 cells was detected by qPCR. **indicates *p* < 0.01; *indicates *p* < 0.05

As expected, AD-MSCs co-cultured with BV2 cells for 24 h (Fig. 7A, *P* < 0.01) and 48 h (Fig. 7B, *P* < 0.01) showed that the former could dramatically down-regulate the proliferation activity of LPS-activated BV2 cells. What’s more, as shown in Fig. 7C, the mRNA level of cyclin D1 (CCND1)in BV2 cells that exposed to AD-MSCs was significantly lower than that in LPS-treated group (*P* < 0.01).

### AD-MSCs inhibited the migration of LPS-activated BV2 cells

**Fig. 8:**
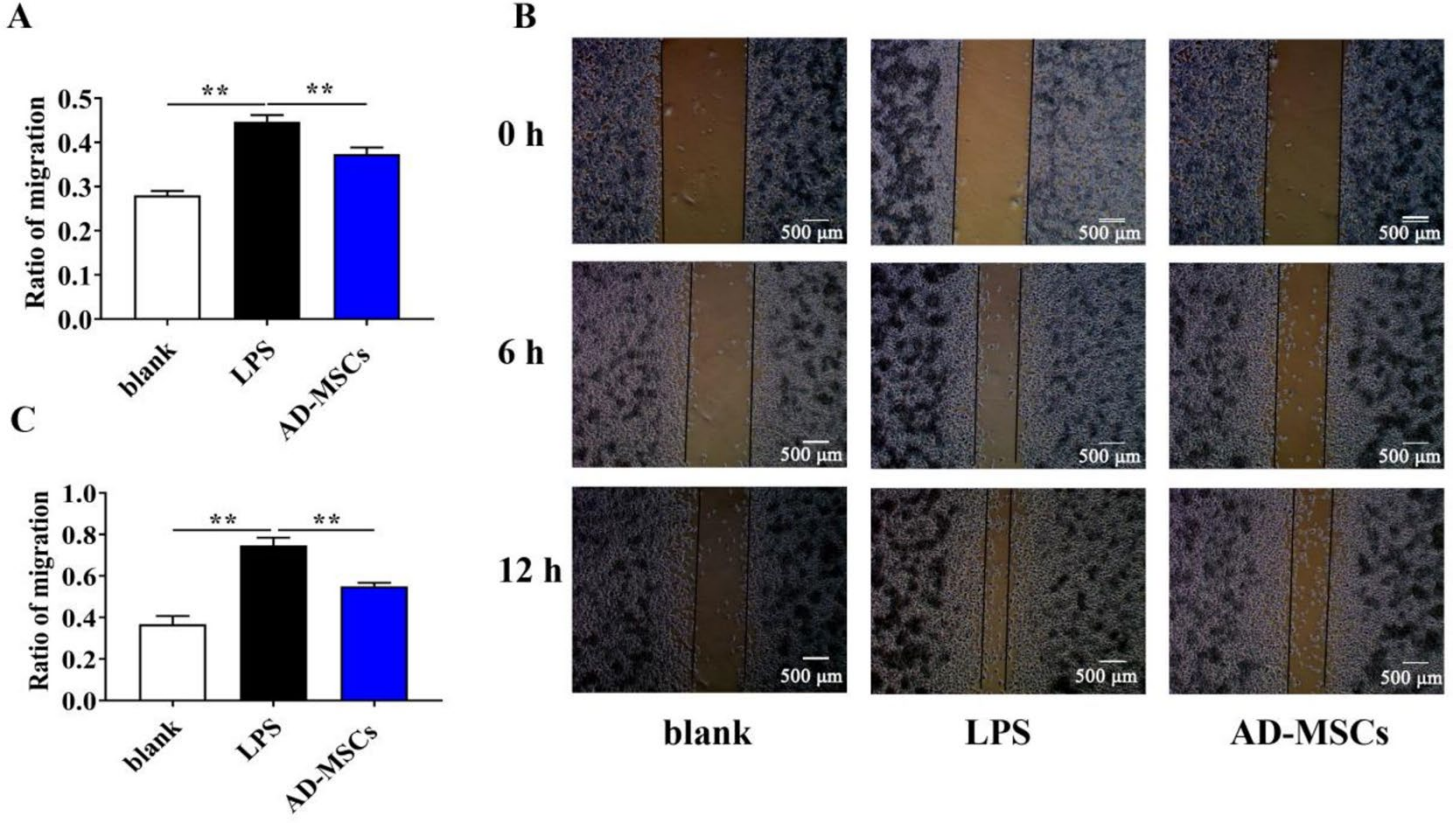
AD-MSCs inhibited the migration of LPS-activated BV2 cells. **A** and **C** The migration photo of BV2 cells at 6 and 12 h were analyzed using Image J software v1.8. **B** The migration photo of BV2 cells. **indicates *p* < 0.01; *indicates *p* < 0.05

In addition to proliferation, reducing microglial migration also plays an important role in the recovery of TBI. We employed scratch wound assay to analyze the effect of AD-MSCs on microglial migration. Results from Fig. 8A, B and C demonstrated that AD-MSCs inhibited the migration of BV2 cells, as compared with LPS-exposed group (*P* < 0.01).

### AD-MSCs can improve TBI-induced neuroinflammatory environment

**Fig. 9:**
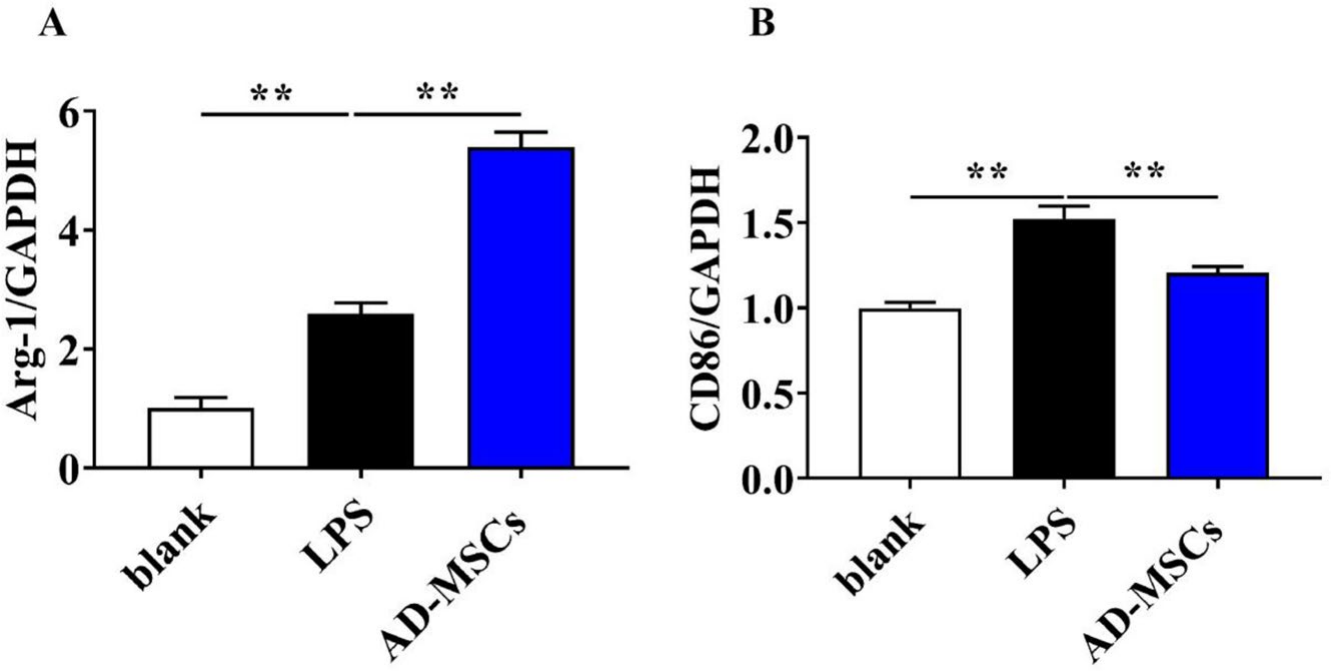
The effect of AD-MSCs on polarization of BV2 cells. **A** and **B** The mRNA expression of Arg-1 and CD86 genes of BV2 cells was detected by qPCR. **indicates *p* < 0.01; *indicates *p* < 0.05

**Fig. 10:**
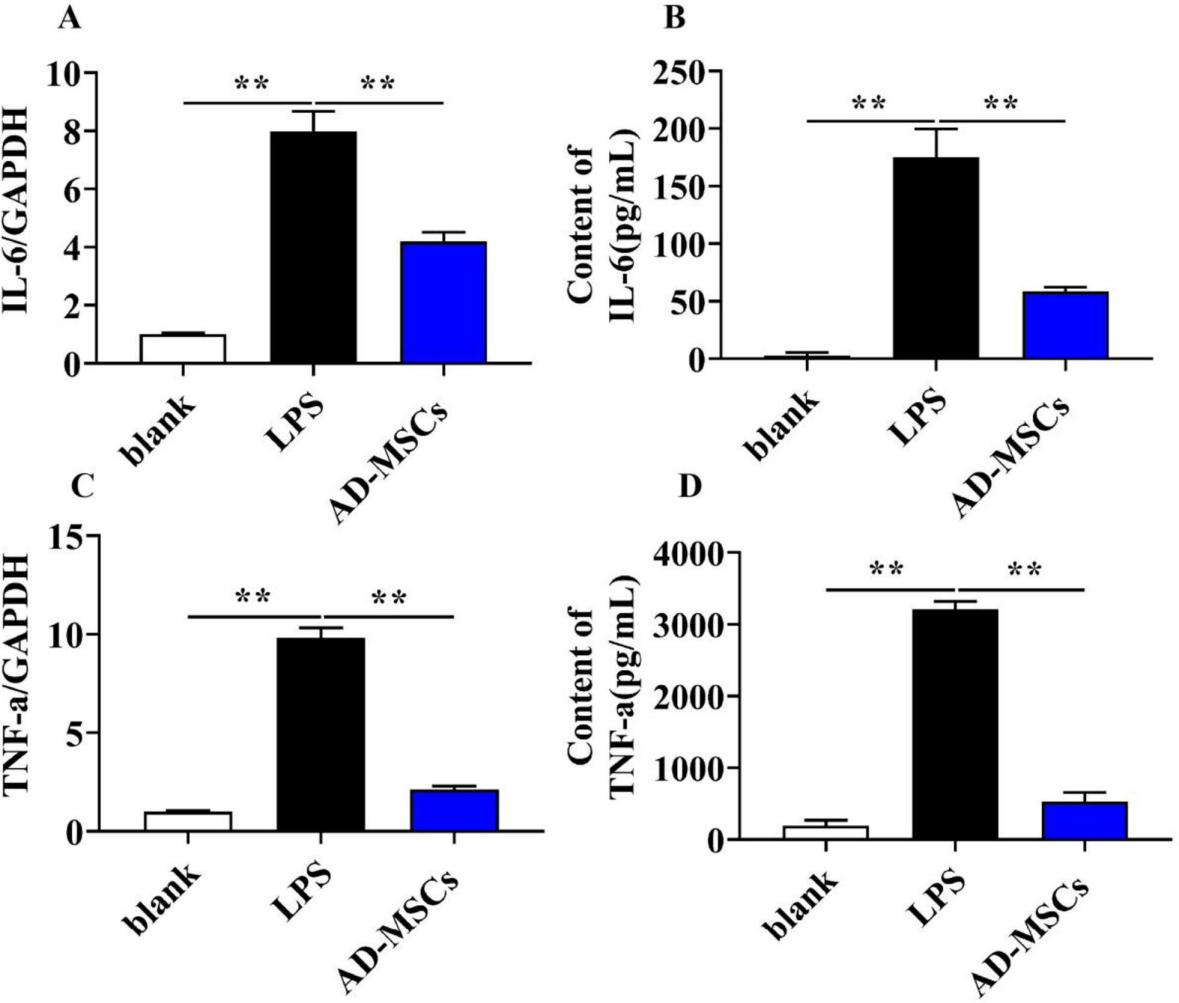
The effect of AD-MSCs on the expression of inflammatory cytokines of BV2 cells. **A** and **C** The mRNA expression of IL-6 and TNF-α genes of BV2 cells were detected by qPCR. **B** and **D** The mRNA expression of IL-6 and TNF-α protein were detected by ELISA. **indicates *p* < 0.01; *indicates *p* < 0.05

CD86-labeled cells were regarded as of the M1 phenotype, and arginase 1(Arg-1)-labeled cells were regarded as of the M2 phenotype. As shown in Fig. 9A and B, the mRNA level of Arg-1 in AD-MSCs with LPS were significantly higher than that in LPS group (*P* < 0.01), and the mRNA level of CD86 in AD-MSCs with LPS with LPS were remarkably lower than that in LPS group (*P* < 0.01), indicating that AD-MSCs are capable of promoting BV2 cell polarization from M1 to M2. Besides, the gene and protein levels of M1-related pro-inflammatory cytokines, IL-6 and TNF-α that secreted by BV2 cells were investigated by q-PCR and Elisa kit. As expected, both the IL-6 and TNF-α expression in BV2 cells were dramatically decreased that exposed to AD-MSCs with LPS (Fig. 10A, B, C and D, *P* < 0.01.), which demonstrated that AD-MSCs have the potential to inhibit LPS-induced BV2 inflammatory response.

### AD-MSCs reduced LPS-induced production of NO by microglial cells

**Fig. 11:**
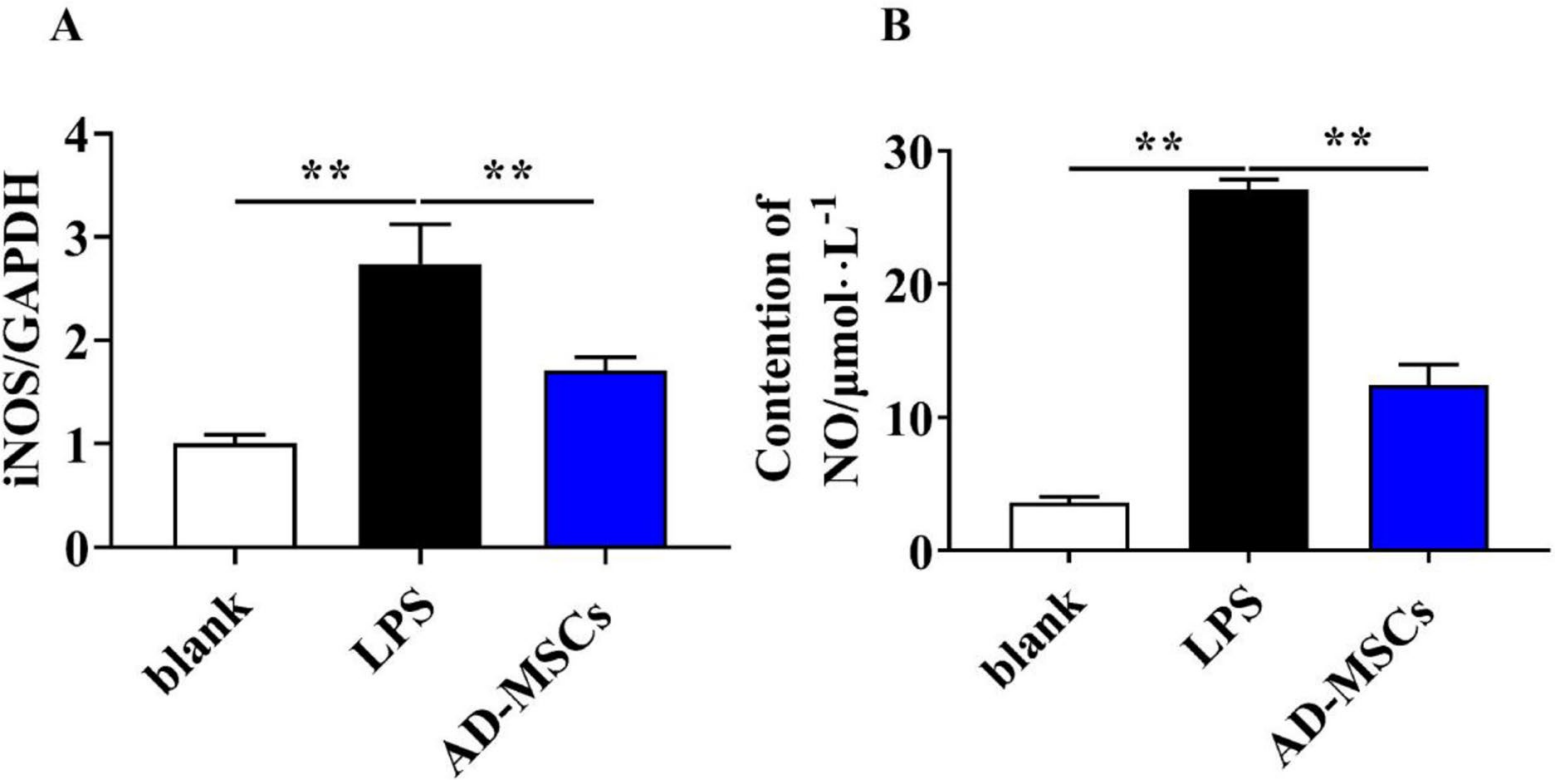
AD-MSCs reduced LPS-induced production of NO by microglial cells. **A** The mRNA expression of iNOS genes of BV2 cells were detected by qPCR. **B** NO secretion level of BV2 cells were detected by Total Nitric Oxide Assay Kit. **indicates *p* < 0.01; *indicates *p* < 0.05

First, we investigated the effects of AD-MSCs on LPS-mediated NO production by microglial cells. After intervention of AD-MSCs for 24 h, NO production was significantly reduced in co-cultures group (Fig. 11A, *P* < 0.01). Besides, the mRNA level of inducible nitric oxide synthase(iNOS) was induced in BV2 cells after 24 h exposure to LPS. A significant reduction in LPS-induced iNOS expression was observed in cultures treated with AD-MSCs co-cultures (Fig. 11B, *P* < 0.01).

## Discussion

TBI is a neurosurgical disease with structural or functional impairment of the brain caused by mechanical force [13]. Due to the complex secondary injury of TBI, there is no effective treatment at present. MSCs possess strong tendency and paracrine ability to secrete a variety of cell factors to the injured tissue. Recent studies have indicated that MSCs could promote neurological function recovery in TBI rats, but the exact mechanism is still not clear [14, 15]. This study demonstrated that canine AD-MSCs promote central nervous system repair by inhibiting neuronal death and gliocyte overactivation in TBI rats.

Neurons are the structural and functional units of the nervous system, which are unable to regenerate after death. The number of surviving neurons directly affects the motor, sensory and cognitive abilities [16]. Secondary injuries such as increased excitatory amino acids, inflammation, oxidative stress and excessive activation of calpain in brain tissue caused by TBI lead to break the balance of Bcl-2/Bax, thus leading to neuronal apoptosis [17]. NeuN is a marker of mature neurons. Our results of immunohistochemistry analysis and real time quantitative PCR showed that the expression of NeuN in AD-MSCs group was significantly higher than that in TBI rats, and the level of Bcl-2/Bax was also increased. According to the results of modified neurological severity scores, the recovery of neurological function was significantly improved after AD-MSCs intervention. From the above results, we speculate that the intervention of AD-MSCs can effectively reduce the apoptosis of neurons in TBI rats, thus promoting the recovery of neurological function in rats.

The main role of astrocytes in the central nervous system is to provide cellular communication and structural support for neurons [18]. However, the overproliferation of astrocytes form glial scarring after brain injury, which is a major obstacle to axonal regeneration and neuronal connection restoration [19]. Upregulation of GFAP expression signals activation of astrocytes. Immunofluorescence analysis and RT-qPCR results showed that AD-MSCs intervention could significantly reduce formation of glial scar in the brain of rats after TBI, reducing barriers to neuronal axonal regeneration.

As the immune cells of the central nervous system, during TBI inflammation, the number of microglia rapidly increases at the injury site to exacerbate inflammation, therefore reducing the proliferation of microglia, which will markedly facilitate the recovery of TBI [20]. BV2 is a microglial cell line, which has the physiological characteristics of primary microglia, thus BV2 cells were used as the in vitro study model of the neuroinflammatory after TBI in rats. The activated microglias are divided into M1 phenotype and M2 phenotype. M1 phenotype microglias promote the release of inflammatory factors and neurotoxins, which is one of the main reasons of neuronal death [21]. In contrast, M2 phenotype microglias are associated with repair of damaged cells and resolution of the inflammatory cascade [22].

It is reported that MSCs could promote activated microglias to polarize into the M2 phenotype and alleviate further pro-inflammatory reactions by releasing anti-inflammatory factors [23]. CD86 is a marker of M1 microglia. Our data showed that the expression of CD86 gene in BV2 cells was significantly down-regulated after co-culture with AD-MSCs. On the contrary, the level of Arg-1 gene, a marker of M2 microglia, was significantly up-regulated. In addition, we found that AD-MSCs could effectively inhibit the proliferation and migration of microglia and reduce the secretion of inflammatory factors TNF-α and IL-6, reflecting the anti-inflammatory capacity of AD-MSCs, which is similar to those of previous studies [24, 25]. It is well known that iNOS is capable of inducing a large amount of NO in the inflammatory response of TBI, so down-regulation of iNOS also has an anti-inflammatory effect [26]. Moreover, NO causes neuronal death by inhibiting mitochondrial respiration [27]. Our study showed that AD-MSCs reduce the production of NO by inhibiting the expression of iNOS gene. Similarly, Zhou et al. also found that bone marrow-derived MSCs can also reduce the secretion of NO and iNOS from PLS-stimulated microglia [28].

## Conclusion

In conclusion, our study demonstrated that canine AD-MSCs could improve the neurological function of TBI rats by inhibiting neuronal death and glial cells hyperactivation.

## Methods

### Materials

MSCs complete medium and DMEM medium was obtained from Cyagen Biosciences (Guangzhou, China). DAPI, Trizol, and Proteinase K were purchased from Beyotime Biotechnology (Shanghai, China). Zoletil and Sumianxin injection were obtained Meizong Biotechnology (Beijing, China). CCK-8 kit was obtained from Dojindo Chemical (Shanghai, China). Other reagents and chemicals used were of analytical or chromatographic grade.

### Animals

Forty-five SPF Wistar rats (from Guangdong Medical Experimental Animal Center: SCXK(YUE) 2018-0002) weighing between 200 and 220 g were maintained in controlled conditions (12/12 light: dark cycle, at 25℃) with ad libitum access to food and water. The present research was approved by the committee for experimental ethics in animal use of Foshan University.

### TBI Model and Groups

Briefly, rats were anesthetized using Zoletil and Sumianxin injection following manufacturer’s instructions and then the head of the animal was fixed on a stereotactic frame. After alternating disinfection with iodophor and alcohol, the skull was exposed by cutting the head skin along the midline of the rat scalp. A 5-mm-sized bone window was opened at 2 mm behind the right coronal suture and 1.5 mm lateral to the sagittal suture of the rats, but the dura was intact. A 4.5-mm-diameter cylinder bar weighing 20 g was thrown down on the brain dura causing TBI. The scalps of the rats were sutured and placed on a 37℃ thermostatic heating pad until they woke up. Sham-operated rats underwent the same procedures as TBI rats except that they were not impinged on the dura of the brain [29].

About 30 min after TBI molded, the rats were randomly divided to three groups (*N* = 15): (1) Sham group was not impinged on the dura of the brain; (2) TBI group was subjected to crush lesion followed by transplantation of saline solution; and (3) AD-MSCs group was subjected to crush lesion followed by transplantation of canine AD-MSCs.

### Canine AD-MSCs isolation, culture and dentification

Method for isolation, culture and dentification of canine AD-MSCs were according to our previous study [30]. Briefly, Approximately 2–6 × 10^6^ AD-MSCs were obtained from canine adipose tissue (*n* = 12, range 2–5 g), digested by type I collagenase and seeded into 100 mm cell -culture dishes. After 5–7 days, the cells grow reached 80% confluence and were passaged with a 1:3 split ratio. The cells were frozen when they were passaged to P3. Cell surface markers of AD-MSCs were identified by flow cytometry, and three-lineage differentiation capacity was assessed by in vitro induction.

### AD-MSCs transplantation

Briefly, rats were anesthetized using Zoletil and Sumianxin injection following manufacturer’s instructions. 24 h after TBI modeled, a hole ( about 1 mm in diameter ) was drilled at 1.1 mm behind the right coronal suture and 1.5 mm lateral to the sagittal suture of the rats and the syringe needle which the outer diameter is 0.21 mm and the inner diameter is 0.11 mm was inserted 4 mm into the rat brain tissue. Then a volume of 20 µL AD-MSCs (1 × 10^6^ cells) and saline solution used as a control were injected at the speed of 1 µL/min to the rats in the AD-MSCs group and the TBI group, respectively. After the injection, the needle was retained for 10 min and the skin was sutured and disinfected using povidone-iodine.

### Measurement of neurological impairment score

Rats were subjected to exercise, walking, sensation, balance, and reflex examinations and assigned a Modified Neurological Severity Score ( mNSS ) method as described previously [31] that was recorded when a task failed to be completed or when the corresponding reflex was lost. After that, neurological scores were calculated to evaluate the therapeutic effect of AD-MSCs on TBI.

### ELISA

Serum samples were collected from 6 rats of each group on the 3rd day of treatment by puncturing the orbital plexus of the eye and stored at − 80 °C before using. The concentrations of IL-6, TNF-α and IL-10 were measured by using commercial ELISA kits following manufacturer’s instructions (NeoBioscience Technology Co., Ltd., Guangzhou, China).

The supernatants from blank group, LPS group (neuroinflammatory model group) and AD-MSCs group were collected and stored at − 80 °C before using. The concentrations of TNF-α and IL-6 were measured by using commercial ELISA kits following manufacturer’s instructions (NeoBioscience Technology Co., Ltd., Guangzhou, China). The concentrations of cytokines in all the samples were higher than the detection limits.

### Realtime quantitative PCR

Brain tissues were collected from 5 rats of each group on the 21st day of treatment and the RNA of the 3 mm injuries tissue was extracted using trizol method. The cDNA was synthesized by using PrimeScriptTM RT reagent Kit (Takara, Kyoto, Japan) following manufacturer’s instructions. The quantitative real-time PCR was done using TB Green PCR Master (Takara, Kyoto, Japan) on PikoReal™ qPCR system (Thermo-Fisher, USA) to determine the mRNA level of GFAP, IBA1, NeuN, Bax, and Bcl-2, using GAPDH as internal control. The mRNA levels were expressed as the ratio between the mRNA level of the target genes and the internal control gene using the comparative 2^−ΔΔCt^ method.

Also, 4 × 10^5^ BV2 cells were seeded in 6-well plates and and cultured for 24 h. Then they were exposed to blank medium as a control, 1 µg/mL LPS, AD-MSCs (2 × 10^5^) with 1 µg/mL LPS, respectively. After 24 h exposure, the RNA extraction, cDNA synthesis, and quantitative real-time PCR were performed in the same way. The mRNA level of CCND1, CD86, iNOS, Arg-1, TNF-α, and IL-6 were determined. The primers are provided in Table 1.

**Table 1:** Primers sequences used for qRT-PCR.

Name	Accession number	Sequences (5′–3′)	Product,bp
GFAP forward	NM_017009.2	AGAGGAAGGTTGAGTCGCTGGAG	145
GFAP reverse		AGAGCCGCTGTGAGGTCTGG	
IBA1 forward	NM_017196.3	AGCGAATGCTGGAGAAACTTGGG	84
IBA1reverse		CCTCGGAGCCACTGGACACC	
NeuN forward	NM_001134498.3	CCACCACTCTCTTGTCCGTT	165
NeuN reverse		ATCAGCAGCCGCATAGACTC	
Bcl-2 forward	NM_016993.2	GAACTGGGGGAGGATTGTGG	80
Bcl-2 reverse		GGGGTGACATCTCCCTGTTG	
Bax forward	NM_017059.2	GTCCTCACTGCCTCACTCAC	189
Bax reverse		GTTTATTGGCACCTCCCCCA	
GAPDH forward	NM_001394060.2	TTCCTACCCCCAATGTATCCG	270
GAPDH reverse		CCACCCTGTTGCTGTATCCATA	
CCND1 forward	NM_001379248.1	GAGGCGGATGAGAACAAGCAGATC	96
CCND1 reverse		GGAGGGTGGGTTGGAAATGAACTTC	
CD86 forward	NM_019388.3	TCTGCCGTGCCCATTTACAAAGG	104
CD86 reverse		TGCCCAAATAGTGCTCGTACAGAAC	
iNOS forward	NM_001313922.1	CAGCTGGGCTGTACAAACCTT	95
iNOS reverse		CATTGGAAGTGAAGCGTTTCG	
Arg-1 forward	NM_007482.3	AACCTTGGCTTGCTTCGGAACTC	131
Arg-1 reverse		GTTCTGTCTGCTTTGCTGTGATGC	
TNF-α forward	NM_001278601.1	ACGCTCTTCTGTCTACTGAACTTCG	113
TNF-α reverse		TGGTTTGTGAGTGTGAGGGTCTG	
IL-6 forward	NM_001314054.1	ACTTCCAGCCAGTTGCCTTCTTG	110
IL-6 reverse		TGGTCTGTTGTGGGTGGTATCCTC	

### HE staining

Twenty-one days after TBI-modeled, 5 rats from each group were anesthetized and were perfused transcardially with saline solution, followed by 4% paraformaldehyde in 0.1 M PBS, pH 7.4. Then, the brain tissue was cut with a scalpel, fixed, dehydrated, embedded, and made into tissue sections with a thickness of 4 mm followed by HE staining and observed under the light microscope.

### Immunofluorescence and immunohistochemistry

Antigen retrieval was carried out and the sections were incubated with 0.3% H_2_O_2_ in PBS for 10 min, treated with 0.3% Triton-X 100 for 20 min and blocked with 1% BSA solution for 20 min. Then they were incubated with primary antibodies GFAP (1:200) or IBA1 (1:200) at 4 °C overnight. After rinsing with PBS three times, sections were incubated with fluorescently labeled secondary antibody at 25 °C for 2 h in the dark, observed and photographed after counterstaining with DAPI.

After treating with 0.3% H_2_O_2,_ 0.3% Triton-X 100 and 1% BSA solution, sections incubated with primary antibodies NeuN (1:200). After rinsing with PBS, sections were incubated with HRP labeled secondary antibodies at 25 °C for 2 h. Each of the steps was followed by three 5-minute rinses in PBS. Nuclei were stained with hematoxylin and then observed under a microscope.

### CCK-8 assays

The cytotoxicity of LPS against BV2 cells was assessed by CCK-8 according to the manufacturer’s instructions. Briefly, BV2 cells were cultured in DMEM medium with 10% FBS and treated with 0, 0.125, 0.25, 0.5, 1, and 2 µg/mL LPS for 24 h. Recommended dosage of CCK-8 reagent was added and incubated for 1 h. Then a microplate reader (Thermo MK3, USA) was used to detect the absorbance at 450 nm.

The effects of canine AD-MSCs on the proliferation of activated BV2 cells were also investigated. Briefly, 1 × 10^5^ BV2 cells were seeded in 24-well plates and cultured 24 h. Then, the blankgroupwere treated with 0.8 mL medium. The LPS group were treated 0.8 mL medium with medium containing 1 µg/mL LPS. AD-MSCs (0.6 × 10^5^) were added in transwell culture chamber for AD-MSCs group, and Other treatments were the same as those in the LPS group. After 24 h cultured, cell proliferation was detected by CCK-8 assays.

### Scratch wound assay

5 × 10^5^ BV2 cells were seeded in 24-well plates and cultured overnight. When the cell density reaches 95%, the monolayer was scratched using a 200 µL pipette tip and detached cells were rinsed out with PBS. Then blank group, LPS group, and AD-MSCs group were exposed to blank medium as a control, 1 µg/mL LPS, AD-MSCs (0.6 × 10^5^) with 1 µg/mL LPS, respectively. The migration of cells was observed under the microscope at 0 h,6 and 12 h, respectively, and photographed.

### Detection of NO release

The supernatants from blank group, LPS group, and AD-MSCs group were collected and stored at − 80 °C before using. Total Nitric Oxide Assay Kit of Beyotime Biotechnology were used to detect the secretion of NO in each group by Griess Reagent method. Experiment procedure according to the instructions.

### Statistics

All experiments were carried out with at least 3 replicates per group. The data shown are representative of these experiments and are presented as the mean ± SD. Multiple group comparisons were performed by two-way analysis of variance with Tukey’s post hoc test. Statistical analysis was conducted using GraphPad Prism 7.0 software, and statistical significance was declared as (*) *p* < 0.05, (**) *p* < 0.01 and (***) *p* < 0.001.

## Data Availability

The datasets used and/or analysed during the current study are available from the corresponding author upon reasonable request.

## Abbreviations

TBI: Traumatic brain injury
AD-MSCs: Adipose-derived mesenchymal stem cells
MSCs: Mesenchymal stem cells
BBB: Blood-brain barrier
mNSS: Modified Neurological Severity Scores
GFAP: Glial fibrillary acidic portein
IBA1: Ionic calcium binds adapter molecule 1
NeuN: Neuronal nuclei
Bax: BCL2-Associated X Protein
Bcl-2: B-cell lymphoma-2
CCND1: Cyclin D1
INOS: Inducible nitric oxide synthase
Arg-1: Arginase 1
LPS: Lipopolysaccharide
CCK-8: Cell counting kit-8
